# Multiple Damage Detection of an Offshore Helideck through the Two-Step Artificial Neural Network Based on the Limited Mode Shape Data

**DOI:** 10.3390/s21217357

**Published:** 2021-11-05

**Authors:** Byungmo Kim, Chanyeong Kim, Seung-Hyun Ha

**Affiliations:** Department of Ocean Engineering, Korea Maritime and Ocean University, Busan 49112, Korea; bmkim@g.kmou.ac.kr (B.K.); odreeblue@g.kmou.ac.kr (C.K.)

**Keywords:** damage detection, artificial neural network, offshore helideck, mode shape prediction, structural integrity assessment

## Abstract

A helideck is an essential structure in an offshore platform, and it is crucial to maintain its structural integrity and detect the occurrence of damage early. Because helidecks usually consist of complex lattice truss members, precise measurements are required for structural health monitoring based on accurate modal parameters. However, available sensors and data acquisition are limited. Therefore, we propose a two-step damage detection process using an artificial neural network. Based on the mode shape database collected from 137,400 damage scenarios by finite element analysis, the neural network in the first step was trained to estimate the mode shapes of the entire helideck model using the selected mode shape data obtained from the limited measuring points. Then, the neural network in the second step is consecutively trained to detect the location and amount of structural damage to individual parts. As a result, it is shown that the proposed procedure provides the damage detection capability with only a quarter of the entire mode shape data, while the estimation accuracy is sufficiently high compared to the single network directly trained using all mode shape data. It was also found that, compared to the network directly trained from the same data, the proposed technique tends to detect minor damages more accurately.

## 1. Introduction

Offshore structures encounter various hazards during long operation periods. In history, hazards sometimes cause serious consequences such as explosions, collapses, or capsizes. Under such situations, offshore helidecks are the final exit that people on the platform can use to escape. Therefore, structural safety is essential. To ensure safety, the first method is to design it to endure the extreme environment safely in the initial stage. Above all, there are some design codes to follow in designing helidecks, such as CAA-CAP-437 [1], DNV-OS-E401 [2], and API-APR-RP-2L [3]. Based on these standards, several studies have proposed safe designs for helidecks. Appropriate design variables to determine a safe helideck were investigated with case studies, and the effectiveness of the design optimization was also proven to save costs and maintain safety [4]. A cantilever-type lightweight helideck design was proposed using topology optimization, and the sectional dimensions of its members were determined by parameter studies [5]. Kim et al. evaluated lightweight design by linear and nonlinear buckling analysis under various loading scenarios with different landing spots and wind directions and concluded that it was safe [6]. Subsequently, to propose a helideck design that has merit in manufacturing and satisfies conservative and persuasive design criteria for customers, a design domain comprising the arranged section numbers within a pool of commercial steel section products was established, and the optimal design was found using a genetic algorithm with safety constraints using the allowable stress criteria and unity check at the same time [7]. The dynamic stability was also verified by linear and nonlinear buckling analyses.

The second method is to check the structural integrity during the maintenance stage. Several different approaches have been applied in various fields, such as aircraft and bridges. For instance, different types of sensors, such as piezoelectric, laser, and electromagnetic sensors, were comprehensively reviewed for aircraft monitoring, and the piezoelectric transducer was reviewed for application [8]. In terms of a bridge, structural health monitoring (SHM) only based on measured sensor data was attempted [9]. Regarding offshore structures, the acceleration responses of the jacket structure, named the Gageocho Ocean Research Station, and its dynamic properties were studied [10]. Then, a finite element model of the jacket structure was developed and updated to mimic its natural frequencies to identify its elastic moduli and the effect of non-structural masses [11]. With the exception of local inspections such as ultrasonic or X-ray techniques, many engineers widely use methods based on sensed signals in time series, such as strains and accelerations in this field. The operational response of ambient vibration in time series and modal parameters from the response, such as natural frequencies and mode shapes, can be effectively utilized to continuously monitor structural integrity and detect damage. In particular, damage to offshore structures generally grows over time. For instance, small cracks due to fatigue, corrosion, and erosion gradually become wider, and, consequently, harm the integrity of the entire structure. The capsize of the Alexander L. Kielland platform is a representative example of this process [12]. At this point, continuous monitoring in maintenance has a strength.

As aforementioned, considering the pivotal role of the helideck on offshore plants, the structural integrity of the helidecks must always be managed, and it is better to find any damage as soon as it occurs. In this respect, a study to detect damage using an artificial neural network (ANN) was performed [13]. This study developed a multi-layer perceptron (MLP) that provides the location and severity of damages from natural frequencies and mode shapes based on the simulation model of a cantilever-type offshore helideck designed by [7]. However, the accuracy of the modal parameters depends on the quality of the measured signals and the meticulous measurement of the entire structure. It is general that a limited number of sensors are installed because more sensors incur higher costs. In addition, missing values often arise because of errors in the measurement system. Therefore, such a circumstance, that is, incomplete modal data, must be taken into consideration when identifying the structural states. In this regard, there is room for further research [13].

Various approaches have been proposed to compensate for the incompletion of the measurement location. From the perspective of conventional structural engineering, for example, model reduction and expansion [14,15,16,17], sub-structuring [18,19], and so on have been proposed [20]. Recently, studies have been conducted to bring machine-learning techniques to the completion of partial mode shapes. Kourehli suggested a method in which an ANN was used to predict structural damage directly from incomplete modal data and applied it to a simple support beam and a three-story plane frame [20]. Kourehli used a least squares support vector machine to propose a strategy of two sequential stages to consummate incomplete mode shapes and then predict damage states [21]. Goh et al. applied an ANN to predict the mode shapes at unmeasured locations of a simple-support slab beam in the first step, evaluated damages in the subsequent step [22], and extended the method and verified it with the experimental results for a single pre-stressed concrete panel [23].

In this study, with reference to [22], simulation-based damage detection using two steps of ANN is proposed. Damage detection was conducted by applying the method to the cantilever-type offshore helideck as a follow-up study of [13]. Therefore, a transfer learning approach is applied to train the ANN in this study, and the pre-trained network of [13] was used as the source model of transfer learning.

## 2. Materials and Methods

### 2.1. Materials

#### 2.1.1. Specification of the Offshore Helideck

**Figure 1:**
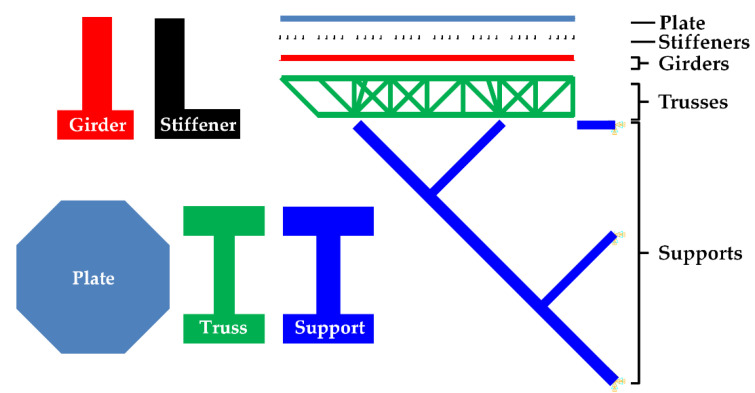
Composition and the cross-sections of the offshore helideck.

The purpose of this study is to apply a two-step damage detection technique to an offshore helideck. The helideck model developed in [7] is used in this study. It consists of a plate, stiffeners, girders, trusses, and supports, and the cross sections are illustrated in Figure 1. The total number of the trusses is 156 and they are divided into three groups, TRS_V_, TRS_H_, and TRH_D_, while a total of 16 support members are categorized into five groups, SPT_1_, SPT_2_, SPT_3_, SPT_4_, and SPT_5_. The members of a group share a section, and the section dimension of each member groups are listed in [7]. The total number of elements of the FE model is more than 80,000. The material is a high-tensile steel HT-36 with a material density of 7850 kg/m^3^ and an elastic modulus of 200 GPa.

#### 2.1.2. Boundary Condition and Intact Mode Shapes

**Figure 2:**
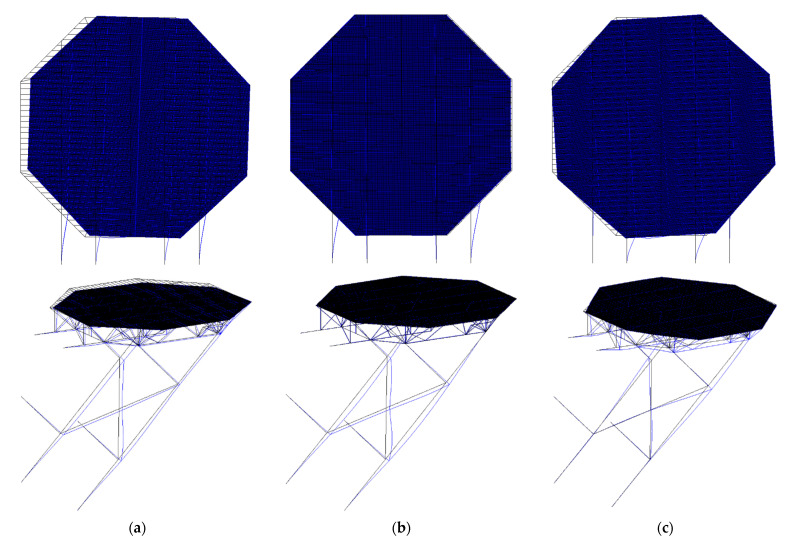
Mode shapes of the intact offshore helideck: (**a**) first mode; (**b**) second mode; (**c**) third mode.

The reference model was an optimal design with the boundary condition that the endpoints of the supports were fixed for every degree of freedom, as shown in Figure 1 [7]. The reason for this is that it is assumed to be connected outwards to an offshore platform, so it should be strongly linked due to the large stresses resulting from the weight of the helideck and due to the importance of the role of the helideck, which is the emergency exit for the workers. Otherwise, it might be split from the main deck and fall into the sea. Therefore, it must be rigidly attached to the main deck. For these reasons, that boundary condition was applied in this study, as well. Under the boundary condition, the mode shapes of the helideck are illustrated in Figure 2, i.e., intact state mode shapes.

### 2.2. Damage of the Helideck

**Figure 3:**
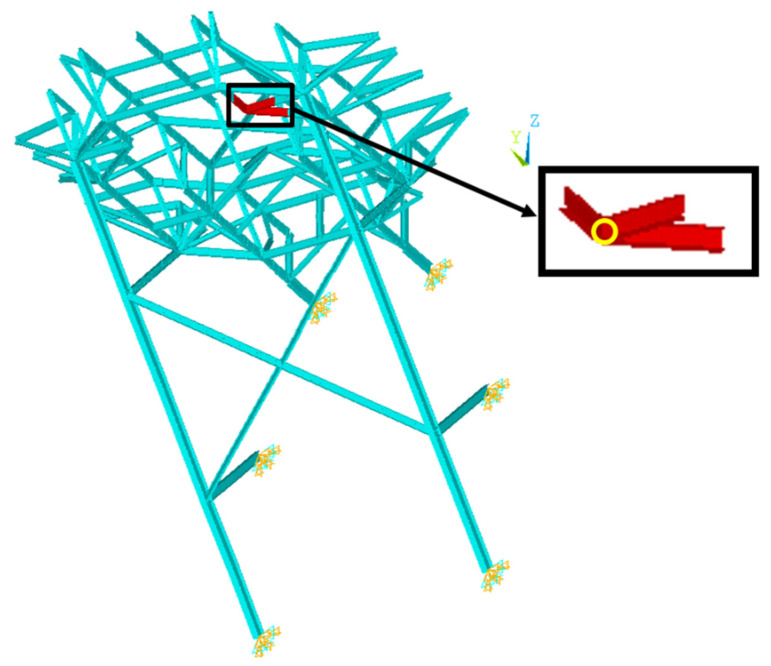
Example of damaged part (Part 18).

This study focuses on the truss and support components for damage detection owing to their roles. Because the trusses bear the weight of the upper deck and the supports connect the helideck to its mother vessel while supporting the whole weight, any critical damage would impact the safety of the helideck as well as its mother vessel. One of the common types of damage to the lattice structure is fatigue, which occurs on joints connected to several members due to various environmental causes, such as corrosion, vortex-induced vibration by wind, and earthquakes, as well as production causes, such as micro-cracks during welding or assembling [18]. Focusing on an offshore structure, the salinity arising from seawater leads steel members to be corroded, and this happens in a wide range over the structure because of droplets of seawater in the air. Additionally, the vibrational response due to waves and winds causes fatigue at the joints. These phenomena not only always occur but also interactively worsen the condition of the structure, i.e., accelerate the degree of damage. Hence, this study deals with that type of damage. With the assumption that such damage weakens the structural capacity of all the members connected to the joint, the truss and support members are grouped into a total of 70 parts based on the intersection joints, and the members in a group are assumed to share the same damage. In Part 18, for instance, there are three red members intersecting at a joint with a circle mark, as shown in Figure 3, and therefore, they would share any damage. The damage was simplified as the decrease in the elastic modulus in this study, because the mechanism of actual physics of the corrosion and fatigue is the matter at the molecular-atomic level, and therefore, it is very difficult to accurately and precisely implement it in a finite element model for a large structure. If the damage severity of this part is assumed to be 10%, the value of the elastic modulus of all five members decreases by 10%.

### 2.3. Procedure and ANN Groups

**Figure 4:**
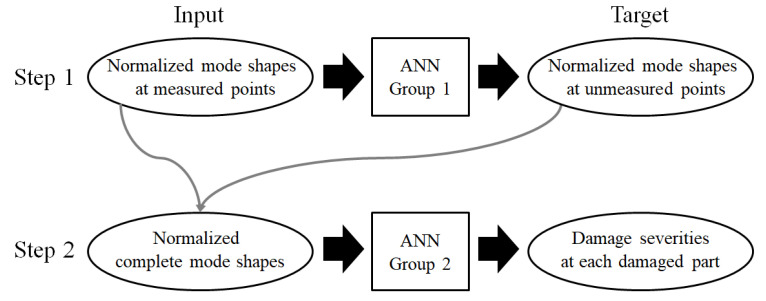
Flowchart of two-step damage detection using ANN.

**Figure 5:**
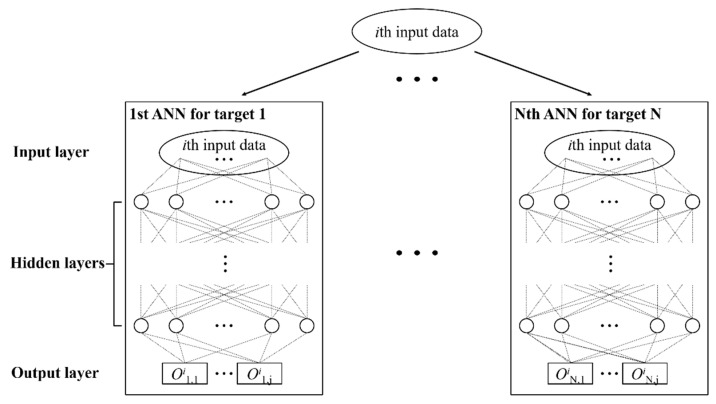
Architecture of an ANN group.

Figure 4 shows a flowchart of the damage detection. The first step is to complete the normalized mode shape of the unknown points. The next step is to evaluate the damage severity of each damaged part. Among 70 neural networks of ANN group 2 in the second step, for example, the neural network corresponding to the group of the red members (Part 21) in Figure 3 predicts the single damage severity of all the elements comprising the red members. Both steps have multiple unknowns to be solved; therefore, ANN groups are constructed in this work to ensure higher accuracy. For example, if there are N targets and a target consists of j elements (i.e., the number of unknown variables is N times j), N single ANNs corresponding to each target are trained to output the predicted values of the target, as shown in Figure 5.

### 2.4. Database for Training ANN

All the data to be used were simulation-based results. Regarding the offshore helideck, 137,400 sets of random damage scenarios were generated depending on the different damage locations. They embraced not only single damage but also multiple damages up to three different locations. Modal analysis was performed for every scenario using ANSYS MAPDL, and then, the corresponding mode shapes for the damage scenario were obtained up to the third modes.

As shown in Figure 4, the damage severity was utilized as a target for ANN group 2. It is the rate of the decrease in elastic modulus, as shown in Equation (1). When damage scenarios were generated, the damage severity was randomly determined within a range of 0 to 0.5. Therefore, it becomes the target of the ANN itself with no modification. For training efficiency, the mode shapes are normalized within the range of −1 to 1, as shown in Equation (2).

(1) $$E_{n}=\frac{E_{n}^{intact}-E_{n}^{damaged}}{E_{n}^{intact}},$$

(2) $$\phi_{k}^{i^{\prime }}=2\times \frac{\phi_{k}^{i}-\phi_{k}^{min}}{\left(\phi_{k}^{max}-\phi_{k}^{min}\right)}-1,$$

where n is the part number out of the total of 70 parts; $E_{n}^{intact}$ and $E_{n}^{damaged}$ are the modulus of elasticity before and after damage of the *n*th part, respectively; $E_{n}$ is the damage severity of the *n*th part; $\phi_{k}^{i}$ is the *k*th element of mode vector of the *i*th data; $\phi_{k}^{min}$ and $\phi_{k}^{max}$ is the minimum and maximum value of the *k*th element of mode vector out of 137,400 data, respectively; and $\phi_{k}^{i^{\prime }\;}$ is the normalized value of $\phi_{k}^{i}$.

Meanwhile, it should be taken into account that the damage may affect the order of the eigenmodes, depending on how severe the damage is. For example, a very local mode that does not appear before damage may arise on the first mode due to damage, or the second mode before damage may become the third after the damage. This confuses the damage detection. In this study, such a matter did not appear in the damage scenarios considering up to three simultaneous damages with the maximum damage severity of 0.5 based on the definition of the damage (Figure 3).

### 2.5. Training Cases

**Figure 6:**
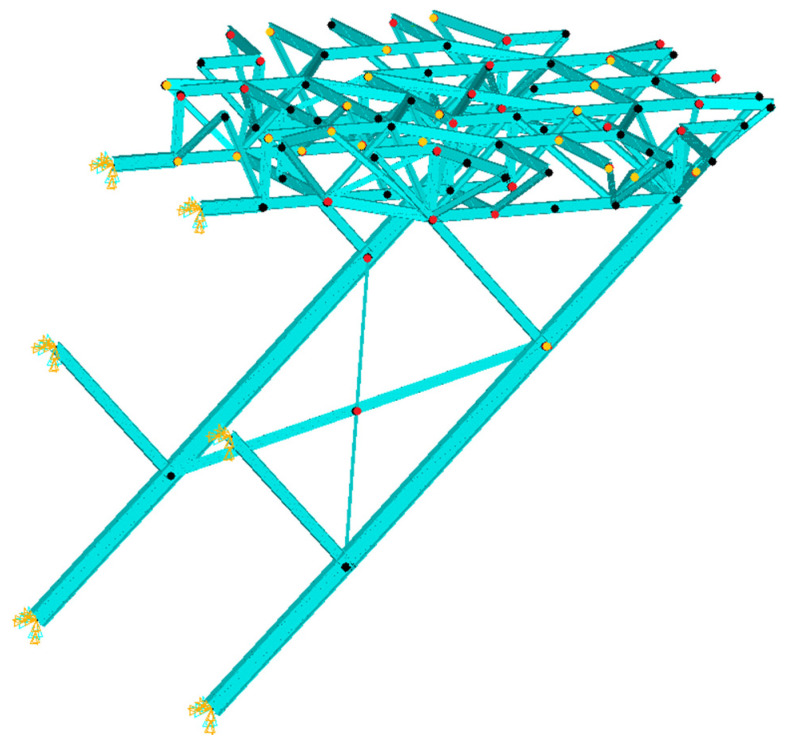
Sensor places (case 3: yellow points; case 2: red and yellow points; case 1: black, red, and yellow points).

A total of 87 measurement points of the truss and support members, including the 70 intersection joints, are used to express the mode shapes of the helideck. In this study, four damage detection cases were trained independently, as shown in Table 1. Case 1 is to identify the damage from the entire normalized mode shapes composed of all 87 points, and therefore, directly goes through step 2 without step 1. On the other hand, it was presumed that only 44 and 22 points were measured for cases 2 and 3, respectively. The sensor places are shown in Figure 6. The yellow points are 22 points of case 3 and the red points added to them are 44 points of case 2. The total of the yellow, red, and black points are 87 points of case 1. The mode shapes at those points are assigned to ANN group 1, and then the mode shapes at the remaining points are estimated through step 1. After finishing the ANN training, the entire mode shapes were collected by assembling the predicted values with the measured values, and the ANN group 2 was trained in the completed mode shapes to predict the damage severities. By comparing cases 1 to 3, the tendency of the results of the proposed damage detection from more limited measurements is observed. Case 4 is the same as case 3, except that it does not go through step 1 to detect damage, and the results of cases 3 and 4 are discussed to comprehend the effectiveness of step 1 when the mode shapes are limited.

### 2.6. Network Structure and Transfer Learning

Because the mode shape of the helideck is defined at 87 points in the 3D model up to the first three modes, the entire mode shapes are expressed as a vector with 783 elements. That is, the number of input data of step 2 is 783, except for training case 4. For case 4, the number of input data is 198. The number of targets in step 2 is the same as the total number of damaged parts, that is, N = 70, as shown in Figure 5. In ANN group 2, therefore, 70 ANNs are separately trained, and each ANN has a single scalar output (j = 1 in Figure 5), which indicates the damage severity of the corresponding part.

In step 1, on the other hand, the numbers of input data are 396 and 198 for training cases 2 and 3, respectively, and the numbers of unidentified data are 387 and 585 for 43 and 65 unmeasured points, respectively. To efficiently train the networks to predict the entire normalized mode shape data precisely, each network of ANN group 1 was set to estimate the 3D mode shape vector which has the three elements (i.e., j = 3) of the normalized XYZ mode shape values for one mode at the unmeasured point. Specifically, the number of targets (N) up to the third mode is 129 and 195 for training cases 2 and 3, respectively; therefore, the respective ANN group in step 1 consists of 129 and 195 neural networks that are independently trained, and each network provides three output data. These results are summarized in Table 2.

The performance in training the ANN depends on the topology of the network and the optimizer used for training; therefore, it takes a long time to execute various attempts to find the best network. Thus, a transfer learning technique has been widely used in many different fields in recent works [24,25,26], to save effort to ensure the high performance of a trained model. In brief, it utilizes a pre-trained network model, in part or whole, to develop a new network to resolve a similar matter. For this reason, this study also applied this approach to efficiency.

**Figure 7:**
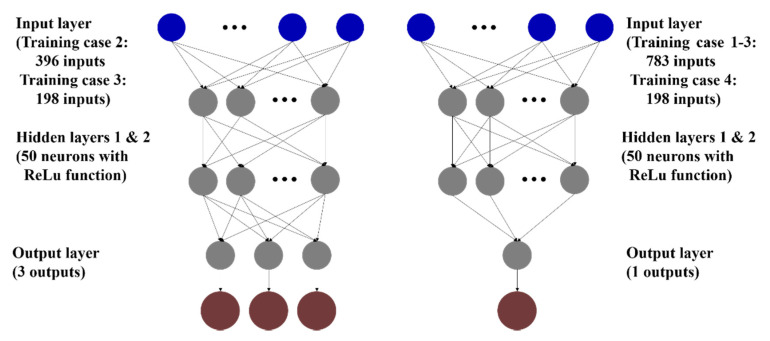
Topology of the networks in ANN groups 1 (**left**) and 2 (**right**).

Yang et al. [26] proposed two transfer strategies for deep learning. The first is to inherit the network architecture together with the weights and biases of the source network model, and hence, it is appropriate for the new dataset to be very similar to the original data; the second is to take only the architecture. In this study, the latter was applied because of the difference between the datasets used in this research and in the source network model developed by [13]. The network consists of two fully connected hidden layers with 50 neurons, without biases. The ReLU activation function was used for each layer. Figure 7 illustrates the topology of a network in ANN groups 1 and 2.

### 2.7. Training Conditions

As a loss function, the mean squared error (*MSE*), which is widely used for regression, is applied in this research as shown in Equation (3).

(3) $$MSE=\frac{1}{M}\sum_{i=1}^{M}{\left(T^{i}-O^{i}\right)}^{2},$$

where M is the number of data and $T^{i}$ and $O^{i}$ are the target and output values of the *i*th data, respectively. To train the networks effectively while preventing overfitting, the data were randomly grouped into three datasets: training, validation, and test datasets. The training dataset was used to update the network weights during iterations of feedforward and backward propagation to reduce the loss function value of the dataset, while the validation dataset was used only to evaluate the loss function at each epoch during the training. The test dataset was not used in training but was used to estimate the performance of the network after completing the network training. The training conditions are presented in Table 3. The maximum number of epochs was 2000. The training is supposed to be stopped before the maximum epochs if the loss for the validation data reaches the minimum loss of 0.000001, or at the number of epochs in which the loss for the validation data does not improve (i.e., the number of validation checks exceeds the maximum validation check of 500). The mini-batch size is determined by considering the size of the data and the capacity of the computer, as shown in Table 4. Training is affected by the optimizer that is applied. Therefore, the adaptive moment estimation (Adam) optimizer was selected because it is one of the most widely used optimizers in recent years and is also known as a convincing method in deep learning.

### 2.8. Estimation of Performance

#### 2.8.1. Modal Assurance Criterion (*MAC*)

The modal assurance criterion (*MAC*) has been widely used since the late 1970s [27] to estimate the similarity of vectors, particularly mode shape vectors. Hence, it was also used in this study to assess how precisely the unmeasured mode shape data were predicted in step 1. It is calculated using Equation (4) [28].

(4) $$MAC\left(r,q\right)=\frac{{\left|{\left\{\phi_{A}\right\}}_{r}^{T}{\left\{\phi_{X}\right\}}_{q}\right|}^{2}}{\left({\left\{\phi_{A}\right\}}_{r}^{T}{\left\{\phi_{A}\right\}}_{r}\right)\left({\left\{\phi_{X}\right\}}_{q}^{T}{\left\{\phi_{X}\right\}}_{q}\right)},$$

where MAC(r,q) is the element of the *MAC* matrix in row r and column q, ${\left\{\phi_{A}\right\}}_{r}$ is the *r*th mode vector of mode shape $\left\{\phi_{A}\right\}$, ${\left\{\phi_{X}\right\}}_{q}$ is the *q*th mode vector of mode shape $\left\{\phi_{X}\right\}$, and the superscript T denotes the transpose of the vector.

#### 2.8.2. Coefficient of Determination

The coefficient of determination ($R^{2}$) is utilized to estimate the prediction performance of the trained networks. It is the ratio of the sum of squared regression (SSR) to the sum of squares total (SST), as shown in Equations (5)–(7). That is, the coefficient indicates how well the predicted values represent the total variation of the real values; therefore, the closer to 1 the value is, the better the outputs agree with the statistical distribution of the targets.

(5) $$R^{2}=\frac{SSR}{SST}\;,$$

(6) $$SSR=\sum {\left(O-\bar{T}\right)}^{2}\;,$$

(7) $$SST=\sum {\left(T-\bar{T}\right)}^{2}\;,$$

where O is the predicted output, T is the target, and $\bar{T}$ is the mean of the targets. Another method is a scatter diagram of the output data versus the corresponding target data. This intuitively explains the regression performance of trained networks.

## 3. Results and Discussion

### 3.1. Loss History

**Figure 8:**
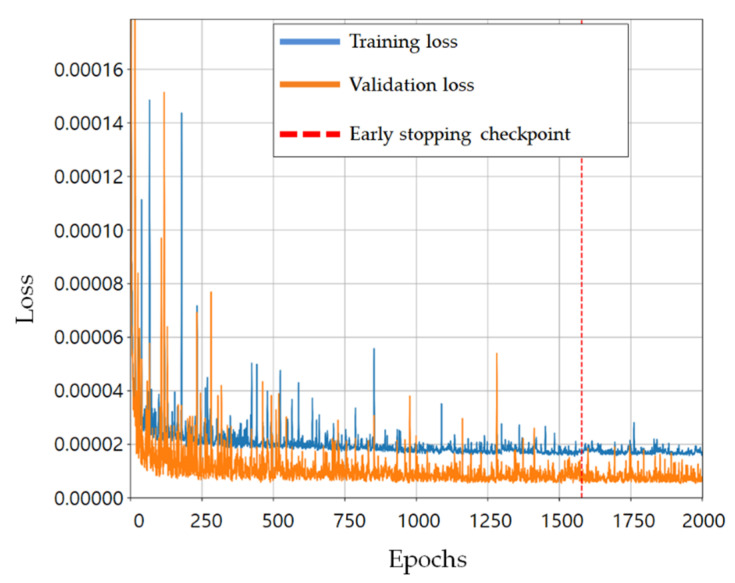
Loss history of the network for damaged part 29 in the training case 1.

Among 137,000 sets in the random damage scenarios, the numbers of training and validation data were 109,600 and 27,400, respectively. As an example, the loss history of the network for damaged part 29 of training case 1 is shown in Figure 8. The blue and orange lines show the losses for the training and validation data, respectively. The training was terminated when the maximum number of epochs was 2000. The vertical red dotted line represents the epoch where the loss function for the validation data is the smallest, and the weight values of the trained network are selected at this epoch to prevent the overfitting problem.

### 3.2. Computational Time for Training Networks

Table 5 lists the time taken to train the ANN groups for the training cases 1–3. Regarding training case 1, approximately 100 h were consumed to train the 70 networks of the ANN group 2, independently. The time to train the ANN group 2 of the training cases 2 and 3 are similar to that because the number of the input data and the target (N = 70) are the same. On the other hand, approximately 255 and 478 h were taken to train the ANN group 1 in training cases 2 and 3, respectively. Although fewer modal data are initially inputted, more time is spent until the completion of the two-step procedure because more networks should be trained to complete the mode shapes. However, the time to predict the mode shapes and identify the damage by the feed-forward propagation is short.

### 3.3. Prediction of Mode Shape Data

As seen in Figure 4, the measured and predicted mode shape data from step 1 are pieced together into the complete normalized mode shapes, which are the inputs of ANN group 2 in step 2. Therefore, the *MAC* matrix of the assembled mode shapes after step 1 and the original mode shapes can be used to estimate the exact description of the assembled mode shapes for the original mode shapes. Because the *MAC* value mathematically means the normalized scalar product of two vectors, as shown in Equation (4), if the predicted mode shapes are equal to the original mode shapes—that is, the unmeasured mode shape data are correctly predicted—the diagonal elements of the *MAC* matrix would become 1. In this study, the *MAC* matrices for all 400 test scenarios were computed, and then averaged to represent the accuracy of the mode shape prediction, as shown in Table 6.

Table 6a,b represents the averaged *MAC* matrix of the original normalized mode shapes and the assembled mode shapes from training cases 2 and 3, respectively, and on the other hand, Table 6c shows the averaged *MAC* matrix between themselves and the original normalized mode shapes. Their diagonal elements are 1, and the matrices are almost identical to one another. This means that the general capability of training cases 2 and 3 for providing the mode shape data at the unmeasured locations is ensured for the test datasets. Therefore, it is proved that it is possible to identify the mode shapes from the limited measurements of a complicated lattice structure. It is also confirmed that the full mode shape can be accurately predicted, even though the number of sensors is significantly limited.

Meanwhile, the off-diagonal elements of the *MAC* matrix from identical mode shapes should be zero because of the orthogonality among the mode vectors of different modes in theory; however, the elements in Table 6a–c are not. In particular, even in the case of Table 6c, the non-diagonal elements of the *MAC* matrix resulting from two identical sets of the original normalized mode shapes are fairly larger than zero, for example, 0.1339. By comparison, the non-diagonal components of the *MAC* matrix calculated from the initial mode shapes before being normalized by Equation (2) for training efficiency are much closer to zero, as shown in Table 6d. Hence, it is observed that the orthogonality of the mode vectors is slightly affected when the data normalization that is generally used before training a neural network for better learning is applied to the database of mode shapes.

### 3.4. Comprehensive Performance of Damage Detection

**Figure 9:**
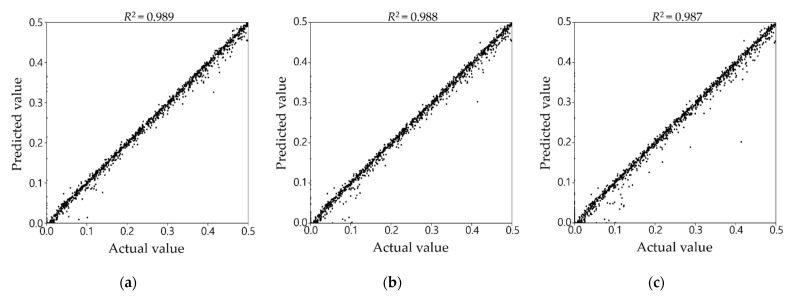
Correspondence of actual versus predicted damage values for the test scenarios: (**a**) training case 1; (**b**) training case 2; (**c**) training case 3.

To verify the effectiveness of the two-step damage detection, the results of training cases 1–3 for all 400 test data were comprehensively compared, as shown in Figure 9. Even with only half and a quarter of the mode shape data used, the R^2^ values through the two-step ANN in Figure 9b,c show little difference from the value in Figure 9a, which is directly obtained from the complete mode shape. A total of 28,000 dots are plotted on each graph in Figure 9, and each dot shows the correspondence between the actual and predicted damage severity. The dot distribution is almost similar to the line of y = x in Figure 9a–c, except for a few outliers. This means that all three networks—that is, not only directly trained from the complete mode shape but also trained through the two-step process from the limited mode shape data—accurately predict the damage severities compared to the actual damage.

Table 7 enumerates the R^2^ values of the three training cases for the test dataset, depending on the individual network by the damaged part. Damaged parts 54 and 61 are the locations where R^2^ is the least in all the training cases; nevertheless, their R^2^ values are greater than 0.9. In most damaged parts, R^2^ of training cases 2 and 3 were almost the same as those of training case 1. This shows that even though only half or a quarter of mode shape data is used, the proposed two-step damage detection technique can provide fairly reliable performance in most parts. In only a few parts, R^2^ is slightly lower because of the limited data. For instance, Part 43 is where the difference in R^2^ among the training cases is the largest. R^2^ in training cases 2 and 3 were approximately 0.054 and 0.082, respectively, less than 0.994 in training case 1. That is, the fewer the mode shape data, the less the R^2^ is. In spite of this, the R^2^ values in training cases 2 and 3 were 0.940 and 0.912, respectively. Consequently, the proposed method was able to accurately detect damage severities with a similar level of precision as the results from the complete mode shapes in most of the damaged parts. In a few parts, although the capability to predict the damage was slightly worse because of the limited sensors, the performance was not significantly worse.

### 3.5. Accuracy of Damage Detection for Test Data

**Figure 10:**
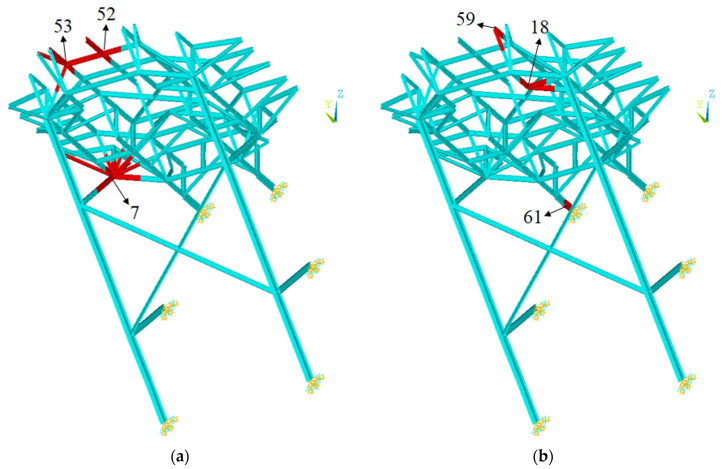
Damaged parts of test scenarios A and B: (**a**) test scenario A; (**b**) test scenario B.

The prediction results for two damage scenarios among 400 test data were compared to show the prediction accuracy of the three training cases in detail. The damage scenarios are shown in Figure 10. Parts 7, 52, and 53 were damaged in test scenario A, while parts 18, 59, and 61 were damaged in test scenario B, in which the prediction error was relatively large among all 400 test cases.

#### 3.5.1. Test Scenario A

**Figure 11:**
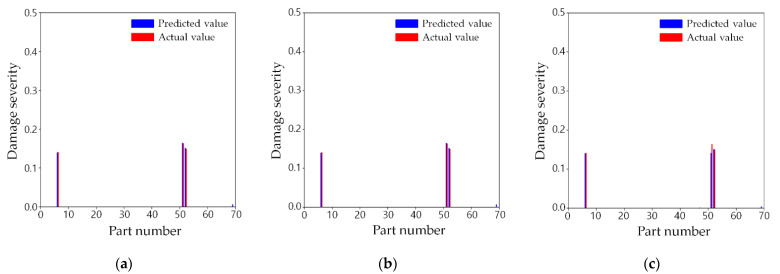
Comparison of predicted and actual damages for test scenario A: (**a**) training case 1; (**b**) training case 2; (**c**) training case 3.

Table 8 shows the actual damage severities of scenario A and the predicted results and errors from the three training cases, whereas Figure 11 compares the predicted damage values versus actual damage severities. First, all the training cases exactly suggest the three damage locations, as shown in Figure 11. The damage severity of Parts 7, 52, and 53 was 0.1412, 0.1637, and 0.1497, respectively. Compared to the prediction result from training case 1, the errors from training case 2 have little difference, even though only half of the measurement points were used. The errors were less than 2% for both training cases. This proved that the proposed method can aid in efficient damage prediction using fewer measurements. Training case 3 also accurately predicted the damage severity within an error of 2%, except for Part 52. The damage severity at this part was underestimated by approximately 0.02 compared to other training cases using much more sensors. Therefore, as the number of measurement points decreased significantly, the accuracy of the damage severity prediction tended to decrease slightly. This approach is still effective considering that only about a quarter of the measurement points were used.

#### 3.5.2. Test Scenario B

**Figure 12:**
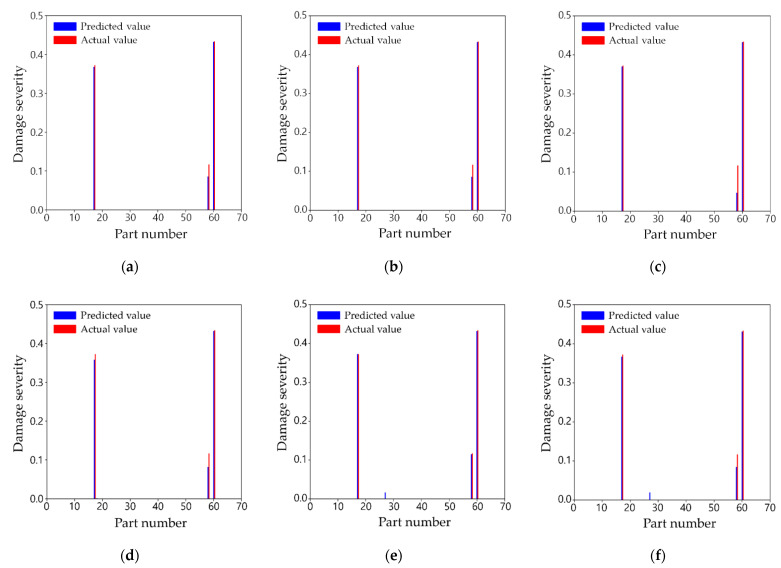
Comparison of predicted and actual damages for test scenario B: (**a**) training case 1; (**b**) training case 2; (**c**) training case 3; (**d**) training case 1 with 1% noise; (**e**) training case 2 with 1% noise; (**f**) training case 3 with 1% noise.

Table 9 shows the actual damage severities of scenario B and the predicted results and errors from the three training cases, and Figure 12a–c shows the predicted damage values versus actual damage severities. The reason for selecting this scenario is that all the three training cases understated the damage severity of Part 59 by about 0.03 to 0.07 than its actual severity. This is probably because the joint connected to this part is a free end far from the fixed points, and the part is composed of only two truss components. This means that damage of Part 59 would affect the modal response less than other parts. In addition, one of the other damaged parts is Part 61, which is the member directly connected to one of the fixed ends. This means that compared to Part 59, the damage of Part 61 would have a much more dominant influence on the dynamic response of the structure. This is supported by the fact that an error of approximately 0.03 occurred even in training case 1, in which all 87 measurement points were used. Nevertheless, the predicted severities of 0.0858 and 0.0872 for this part were calculated from training cases 1 and 2, respectively, and the accuracy showed little difference from each other. With respect to training case 3, the damage severity was further underestimated compared to other training cases. This agrees with the fact in the previous test scenario A, in which much fewer sensors were used, the severity is slightly less accurately predicted. Moreover, it is found from this scenario that the trend grows in a less sensitive location where the decrease in elastic modulus does not induce a notable change in mode shape on the whole. However, the two-step damage detection technique provided the damage part locations and predicted the correct severities in most cases.

On the other hand, the mode shape data, in reality, is computed by applying operational modal analysis techniques to the measured signals in the time domain, and therefore, they are usually a little polluted because the raw signals include noises. Although such an effect was not considered in the mode shape data used in this research, a simple test was carried out to check the possibility of this technique for that matter. Assuming that the effect of the noises follows the normal distribution of which the standard deviation is 1% of the standard deviation of the normalized mode shape data of test scenario B, the polluted mode shape data were calculated by the sum of the random data and the original data, and then inputted to the ANN groups of the training cases 1–3. As shown in Figure 12d–f, the trained ANN groups predicted well where and how the damage occurs despite few contaminated mode shape data, although it is noticeable that one part without damage was misjudged as damage. Hence, this approach enables the diagnosis of damage with a little error, but further research is required to guarantee better accuracy with noise.

#### 3.5.3. Other Test Scenarios

**Figure 13:**
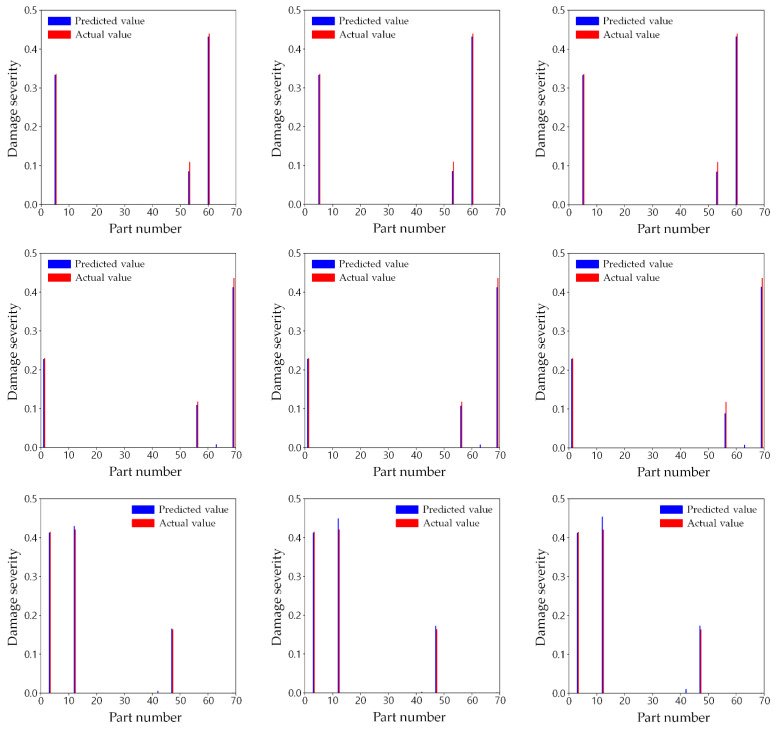

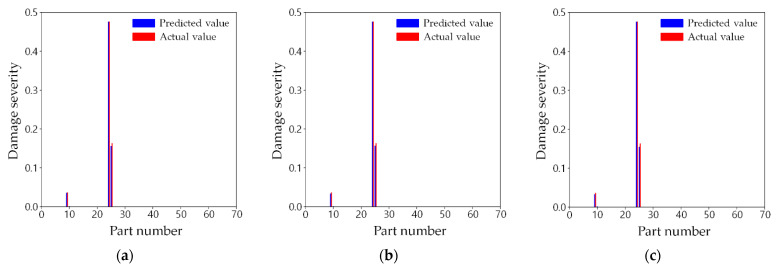
Comparison of predicted and actual damages for four other test scenarios in (**a**) training case 1; (**b**) training case 2; (**c**) training case 3.

To show the general prediction accuracy of the proposed method, Figure 13 illustrates the predicted results of the three training cases for four additional damage scenarios arbitrarily chosen from the other test datasets. Despite the limited number of sensors used, training cases 2 and 3 can determine which parts are damaged and estimate the extent of damage in those parts. In particular, the test scenario in the first row in Figure 13 have three damaged parts and one of them is directly connected to one of the fixed points. Nonetheless, all the damaged parts and their damage severities were found quite accurately as the test scenario B. Therefore, it is convincing that the proposed method enables the prediction of the damage, regardless of how much the damaged parts have a dominant effect on the response of the structure.

### 3.6. Two-Step versus Direct Damage Detection from the Limited Mode Shape Data

**Figure 14:**
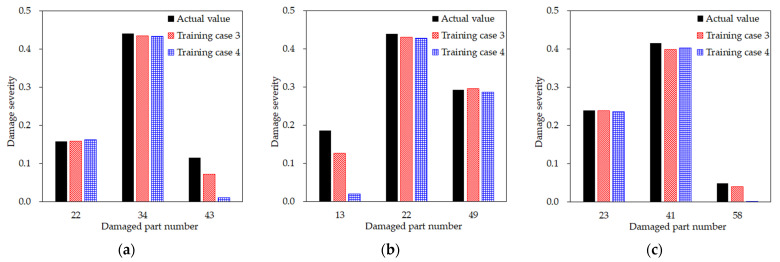

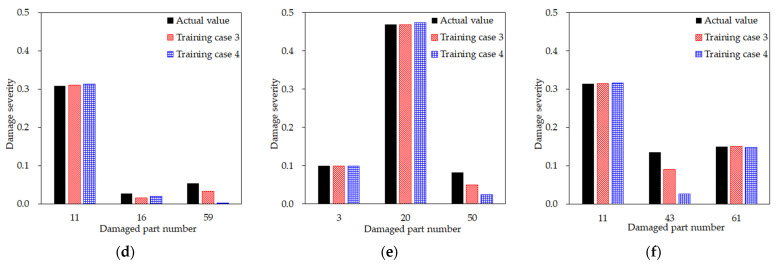
Actual damage for six different test scenarios, versus predicted damage from training cases 3 and 4: (**a**–**f**) different test scenarios.

To verify the effectiveness of the two-step procedure, the prediction results of training cases 3 and 4 for certain test scenarios were compared. As shown in Figure 14, test scenarios were selected in which both training cases correctly located the damage, but the damage severity was not estimated accurately. The first observed finding is that one among the three damages is underestimated. Second, understated damage is not the most severe damage in the three damages, which is less than 0.2. The reason might be that such minor damage is less likely to affect the modal properties compared to other major damages, and therefore, they would be rather difficult to detect. Nevertheless, it is important that training case 3 more correctly identifies the damage severity of the minor damage than training case 4. For example, while training case 4 predicted the severity to be much smaller than the actual damage, training case 3 estimated a much closer value—specifically, over half of the true value. This tendency appears not only in these scenarios. Therefore, it can be concluded that, although it may be unavoidable that very few measurements lead to the underestimation of minor damage under the multiple damage conditions, the proposed two-step technique is advantageous because it can identify relatively less critical but more elusive damage than detecting damage directly with only available sensor data.

## 4. Conclusions

In structural damage detection, it is ideal to acquire structural data at every position of a structure. However, in situ measurements mostly capture limited data at some limited positions. Therefore, a two-step process with individual artificial neural networks was proposed in this research to develop a method of damage detection applied to a complicated truss structure of a cantilever-type offshore helideck based on the limited measurement data. As a result, it was possible to predict the entire mode shape data of the structure from the simulation database and to detect the place and the severity of the multiple damages with high accuracy.

Compared to training case 1 as the reference study, in which the damage was directly detected from the entire mode shape at 87 measurement points, the training cases 2 and 3 that use two-step artificial neural networks provide a reasonable estimation of structural damage from the limited data at only 44 and 22 measurement points, respectively. Therefore, it can be concluded that the proposed two-step method has a similar level of comprehensive prediction performance to the direct prediction with the entire mode shape data, considering that only half or a quarter of the mode shape data were used. It was also found out that the proposed method is applicable for the contaminated mode shape data with numerical noise in the measurements. In this research, mode shape change was not observed from the suggested damage level, but this might be a potential issue when more severe damage is considered.

In future work, this approach can be studied in detecting more severe damage, jumbling the order of the mode shapes and using a smaller number of measurements with the optimal arrangement of sensors. It can also be applied to other complex structures, such as a derrick tower and a flare boom, or a jacket-type substructure of an offshore wind turbine.

## Figures and Tables

**Table 1:** Training cases and the number of measured and unmeasured points of them.

Training Case	Step 1	Step 2	Measured Points	Unmeasured Points
1	X	O	87	0
2	O	O	44	33
3	O	O	22	65
4	X	O	22	65

**Table 2:** Numbers of inputs, outputs, and targets in ANN groups for each training case.

Training Case	ANN Group 1 for Step 1			ANN Group 2 for Step 2		
	Input	Output (j)	Target (N)	Input	Output	Target (N)
1	-	-	-	783	1	70
2	396	3	129	783	1	70
3	198	3	195	783	1	70
4	-	-	-	198	1	70

**Table 3:** Training conditions.

Conditions	Setting
Loss function	*MSE*
Maximum epochs	2000
Minimum loss	0.000001
Maximum validation checks	500
Mini-batch size	512
Optimizer	Adam

**Table 4:** Specifications of the computer.

Component	Specification
CPU	Xeon 6254 3.1 GHz (2EA)
RAM	DDR4 320 GB
GPU	RTX 2080Ti 11 GB

**Table 5:** Computational time for the training cases 1–3.

Training Case	ANN Group	Time for Training
1	2	99.85 h (4.16 days)
2	1	255.47 h (10.64 days)
3	1	478.40 h (19.93 days)

**Table 6:** Averaged *MAC*: (**a**) for the complete normalized mode shapes of the predicted mode shapes from training case 2; (**b**) for the complete normalized mode shapes of the predicted mode shapes from training case 3; (**c**) of the complete normalized mode shapes and themselves; (**d**) of the complete initial mode shapes before normalization and themselves.

	(a) Training Case 2			(b) Training Case 3			(c) Normalized			(d) Initial		
	1	2	3	1	2	3	1	2	3	1	2	3
**1**	1.0000	0.0229	0.1340	1.0000	0.0229	0.1339	1.0000	0.0229	0.1339	1.0000	0.0030	0.0058
**2**	0.0229	1.0000	0.0676	0.0229	1.0000	0.0676	0.0229	1.0000	0.0676	0.0030	1.0000	0.0257
**3**	0.1339	0.0675	1.0000	0.1339	0.0675	1.0000	0.1339	0.0676	1.0000	0.0058	0.0257	1.0000

**Table 7:** R^2^ values for the test dataset of the individual network by damaged part: (**a**) training case 1, (**b**) training case 2, and (**c**) training case 3.

Part No.	(a)	(b)	(c)	Part No.	(a)	(b)	(c)
1	1	1	1	36	1	0.999	0.999
2	1	1	0.999	37	1	1	1
3	1	1	1	38	1	1	1
4	1	1	1	39	0.998	0.998	0.998
5	1	1	1	40	0.964	0.964	0.965
6	1	1	1	41	0.999	0.999	0.999
7	1	1	1	42	0.999	0.999	0.999
8	1	1	1	43	0.994	0.940	0.912
9	1	1	1	44	0.997	0.997	0.997
10	1	1	1	45	0.94	0.939	0.939
11	1	1	1	46	0.999	0.999	0.998
12	1	1	1	47	0.999	0.999	0.999
13	0.997	0.964	0.951	48	1	0.999	0.998
14	1	1	0.999	49	0.984	0.984	0.984
15	1	1	1	50	0.999	0.998	0.998
16	1	1	1	51	0.913	0.913	0.913
17	1	1	1	52	0.994	0.992	0.982
18	1	0.999	0.998	53	0.998	0.998	0.998
19	1	1	1	54	0.904	0.904	0.901
20	1	1	1	55	0.999	0.999	0.999
21	1	1	1	56	0.998	0.998	0.998
22	1	1	1	57	0.985	0.981	0.949
23	1	1	1	58	0.998	0.998	0.998
24	0.998	0.998	0.998	59	0.995	0.994	0.985
25	1	1	1	60	0.999	0.999	0.999
26	0.999	0.999	0.999	61	0.902	0.902	0.902
27	1	1	1	62	0.954	0.954	0.953
28	0.998	0.997	0.998	63	0.953	0.952	0.953
29	0.999	0.999	0.999	64	0.997	0.997	0.997
30	1	1	0.999	65	0.994	0.974	0.974
31	1	1	1	66	0.998	0.998	0.998
32	0.999	0.999	0.999	67	0.996	0.996	0.995
33	0.997	0.997	0.997	68	0.998	0.997	0.998
34	0.999	0.999	0.999	69	0.997	0.995	0.992
35	0.999	0.999	0.999	70	0.987	0.987	0.991

**Table 8:** Actual damage severities of test scenario A and predicted results (and error rate): (**a**) training case 1; (**b**) training case 2; (**c**) training case 3.

Damaged Part No.	Severity	(a)	(b)	(c)
7	0.1412	0.1400 (−0.850%)	0.1399 (−0.921%)	0.1403 (−0.637%)
52	0.1637	0.1645 (+0.489%)	0.1655 (+1.100%)	0.1411 (−13.81%)
53	0.1497	0.1519 (+1.470%)	0.1522 (+1.670%)	0.1513 (+1.069%)

**Table 9:** Actual damage severities of test scenario B and predicted results (and error rate): (**a**) training case 1; (**b**) training case 2; (**c**) training case 3.

Damaged Part No.	Severity	(a)	(b)	(c)
18	0.3725	0.3682 (−0.160%)	0.3677 (−1.289%)	0.3701 (−0.644%)
59	0.1169	0.0858 (−26.60%)	0.0872 (−25.41%)	0.0470 (−59.79%)
61	0.4339	0.4324 (−0.346%)	0.4326 (−0.300%)	0.4323 (−0.369%)
